# Do Standardised Prognostic Algorithms Reflect Local Practice? Application of EORTC Risk Tables for Non-Muscle Invasive (pTa/pT1) Bladder Cancer Recurrence and Progression in a Local Cohort

**DOI:** 10.1100/tsw.2011.77

**Published:** 2011-04-05

**Authors:** Rajiv Pillai, Duolao Wang, Erik K. Mayer, Paul Abel

**Affiliations:** ^1^ Department of Urology, West Suffolk Hospital, Cambridge University Teaching Hospitals Trust, Suffolk, UK; ^2^ Medical Statistical Unit, School of Hygiene and Tropical Medicine, London, UK; ^3^ Division of Surgery, Department of Surgery and Cancer, Imperial College London, St Mary’s Hospital, London, UK; ^4^ Division of Surgery, Department of Surgery and Cancer, Hammersmith Campus, Imperial College Faculty of Medicine, London, UK

**Keywords:** pTa/pT1 bladder cancer, EORTC risk tables, recurrence, progression

## Abstract

A risk calculator algorithm to allow prediction of probabilities of 1- and 5-year recurrence and progression rates in individuals with pTa/pT1 bladder cancer has been proposed by the European Organisation for Research and Treatment of Cancer (EORTC) and was incorporated into the European Association of Urology guidelines in 2006. We attempted to validate this algorithm in a cohort of patients with known outcome. Prognostic data were collected from a consecutively presenting cohort of 109 patients with non-muscle invasive (pTa/pT1) transitional cell cancer (TCC) at a single institution between 1983 and 1985. Using the same statistical models as in the EORTC original paper, predicted probabilities of 1- and 5-year recurrence and progression were calculated. Patients were divided into four risk groups for recurrence (Ir-IVr) and progression (Ip-IVp), respectively, using six prognostic criteria. These were then compared to the probabilities predicted in the EORTC algorithm. The predicted 1- and 5-year probabilities of recurrence were significantly higher in the study population as compared to the original EORTC algorithm for all four risk groups. The predicted 1-year probabilities for progression in groups Ip/IIIp and at 5-years for groups Ip/IIp were in accordance with the original algorithm, but were higher for the other progression groups. The concordance for the model of prediction using the study group for recurrence at 1 and 5 years was 62 and 63%, respectively, and for progression was 65 and 67, respectively. We were unable to validate the proposed algorithm in our group of patients. Although our study has limitations that prevent firm conclusions on the validity of the algorithm, it does expose some of the drawbacks of standardised nomograms when applied to local clinical practice.

## INTRODUCTION

Prediction of outcome in patients with transitional cell cancer (TCC) of the bladder would markedly enhance clinical ability to tailor treatment to individuals, but this goal remains elusive. Prognostic parameters in non–muscle invasive TCC have been the focus of several publications[1,2,3,4,5], but none are sufficiently robust to be useful in routine clinical practice.

In 2007, a risk calculator table based on six prognostic factors, including tumour size, number, pT category, grade, presence of *in situ* disease, and prior recurrence rate, was developed to predict recurrence and progression in individual patients with non–muscle invasive bladder cancer[6]. Data were extracted from a combined analysis of 2596 patients recruited to seven clinical trials undertaken by the genitourinary group of the European Organisation for Research and Treatment of Cancer (EORTC)[6]. The data were used to devise simple tables that would help to predict 1- and 5-year probability of recurrence and progression in individuals. Such tables may usefully be validated in different cohorts of population from different countries and urological institutions before entering routine clinical use.

Our current study reports an attempt to validate the EORTC risk calculator tables using a consecutively presenting cohort of patients with non–muscle invasive bladder cancer and known 5-year outcome derived from a single institution in England.

## MATERIALS AND METHODS

For our study, data were prospectively collected over a 5-year period from a cohort of patients who underwent consecutive transurethral resection (TUR) for new and recurrent bladder tumours at a single institution between April 1983 and February 1985. All patients included in the study had pTa/pT1 TCC as determined by formal histopathological examination. This cohort has been described previously[7]. This prospective data collection provided actual (observed) recurrence and progression rates at 1 and 5 years.

As previously described by Sylvester et al.[6], six clinical and pathological variables used to develop the EORTC algorithm were pT category, grade, size, number, prior recurrence rate, and presence of CIS (carcinoma *in situ*). These variables were derived using a univariate and multivariate analysis of 2596 superficial bladder cancer patients from seven EORTC trials. A weight (score) for each variable was obtained based on the coefficients of variables in the multivariate model. This allowed us to assign the individual patients in our study group a score for both recurrence and progression based on the presence or absence of these six prognostic variables. Based on the score, the patient was allocated to one of the four recurrence (Ir-IVr) and one of the four progression risk (Ip-IVp) subgroups previously defined by Sylvester et al. (Table 1). For example, a patient with a final recurrence score of five and a final progression score of five was assigned into the IIIr recurrence and IIp progression groups, respectively.

**TABLE 1**
**Patient Groups as per Final Recurrence and Progression Scores in the Study Population**

| Recurrence Groups | Recurrence Score | No.of Patients (n = 109) | Progression Groups | Progression Score | No. of Patients (n = 109) |
|---|---|---|---|---|---|
| Ir | 0 | 2 | Ip | 0 | 27 |
| IIr | 1–4 | 68 | IIp | 2–6 | 53 |
| IIIr | 5–9 | 33 | IIIp | 7–13 | 26 |
| IVr | 10–17 | 6 | IVp | 14–23 | 3 |

Using the same methodology as Sylvester et al., we then performed, using our study population, univariate analysis to identify prognostic clinical and pathological variables for recurrence and progression. Multivariate analysis, incorporating these prognostic variables, then generated predictive probabilities allowing direct comparison with the study population's observed 1- and 5-year recurrence and progression rates, and comparison with the predictive probabilities generated using the original EORTC trial data. In this manner, we were able to validate the EORTC nomogram, both in terms of its predictive probabilities and identified prognostic clinical and pathological variables.

Death before recurrence and progression was analysed as a competing risk. Three patients were lost to follow-up during the 5-year period. These were censored at their last documented cystoscopy and therefore included in the analysis.

## Statistics

The Kaplan-Meier method[8] was employed to calculate predicted probabilities of recurrence and progression at 1 and 5 years, respectively. Harrell's bias corrected concordance (c) index[9] ($0 \leq c \leq 1$) was calculated to assess the accuracy of predictions. The c index is a measure of the discriminatory power of the equation ranging from 0.5 to 1.0, with 1.0 as perfect and 0.5 as no better than chance. A Cox regression model was employed to identify the prognostic factors of recurrence and progression. All statistical analysis was performed using SAS 8.2 and R 2.2.0 software

## RESULTS

Between April 1983 and February 1985, 173 patients underwent TUR for bladder tumours. After histological assessment to select only patients with pTa/pT1 TCC, our final study population totalled 109 patients. Median follow-up was 5 years. Table 2 gives the demographic and clinical characteristics of this study group and compares them with the previously reported EORTC group[6]. Ninety-five patients had complete 5-year follow-up data. Of the remaining 14 patients, 11 died and three were lost to follow-up. The causes of death were: medical causes (n = 3), bladder cancer (n = 2), ruptured aortic aneurysm (n = 1), carcinomatosis of unknown origin (n = 1), and unknown (n = 4).

The proportion of patients with recurrence and progression at 5 years was 63.3 and 12.8%, respectively, with a median time to first recurrence of 10 months and a median time to first progression of 60 months. For recurrence, the majority of patients were assigned either to risk groups II or III, whereas for progression, there were relatively equal numbers in risk group I and III, with the highest proportion in risk group II (Table 1). The observed proportion of patients who had recurrence at 5 years increased across the risk groups (I to IV). A similar trend was seen for observed 5-year progression (Table 3). The predicted probabilities for recurrence in all groups of the study population were higher than the EORTC predictions (Table 4). For progression, group IVp showed high progression rates, whereas groups Ip and IIp had comparable results to the EORTC algorithm. Group IIIp had comparable 1-year, but higher 5-year progression rates to the EORTC algorithm.

Figs.1 and 2 show the Kaplan-Meier plots for time to first recurrence and time to first progression, respectively, for the study group, stratified according to the EORTC defined risk groups.

The c index for recurrence was 0.62 for 1 year and 0.63 for 5 years. The c index for progression was 0.65 and 0.67 for 1 and 5 years, respectively.

Univariate and multivariate Cox regression analysis results are presented in Table 5. Number of tumours (>8), tumour size (≥3cm), T category (T1), and CIS (present) were identified as independent prognostic factors in our cohort. For example, the hazard ratio of recurrence and progression for a patient with a number of tumours >8 is 10.89 (95% CI: 2.40, 49.38) times that for a patient with a number of tumours of ≤8.

**TABLE 2**
**Patient Characteristics in Our Study Group and the EORTC Group**

| | Study Group No. (%) | EORTC Group No. (%) |
|---|---|---|
| Total number of patients | 109 | 2596 |
| Age | | |
| <60 | 29 (26.6) | 859 (33.1) |
| 61–70 | 35 (32.1) | 890 (34.3) |
| 71-80 | 31 (28.4) | 690 (26.6) |
| >80 | 13 (11.9 | 118 (4.5) |
| Unknown | 1 (0.9) | 39 (1.5) |
| Gender | | |
| Male | 84 (77.1) | 2044 (78.7) |
| Female | 25 (22.9) | 515 (19.8) |
| Intravesical treatment with single-dose mitomycin | | |
| Yes | 21 (19.3) | 2035 (78.4) |
| No | 88 (80.7) | 561 (21.6) |
| Prior recurrence | | |
| Primary | 103 (94.4) | 1405 (54.1) |
| Prior recurrence (≤1 rec/year) | 3 (2.7) | 505 (19.5) |
| Prior recurrence (>1 rec/year) | 3 (2.7) | 645 (24.8) |
| Number of tumours | | |
| Single | 64 (58.7) | 1465 (56.4) |
| 2–7 | 32 (29.3) | 836 (32.2) |
| ≥8 | 13 (11.9) | 255 (9.8) |
| Size of tumours | | |
| <1 cm | 28 (25.6) | 920 (35.4) |
| <3 cm | 36 (33.0) | 1167 (45.0) |
| ≥3cm | 43 (39.4) | 464 (17.9) |
| Unknown | 2 (1.8) | 45 (1.7) |
| PT category | | |
| Ta | 78 (71.5) | 1451 (55.9) |
| T1 | 31 (28.5) | 1108 (42.7) |
| Presence of CIS | | |
| No | 100 (91.7) | 2440 (94.0) |
| Yes | 9 (8.3) | 113 (4.4) |
| Grade of tumour | | |
| G1/G2 | 98 (89.9) | 2260 (87.1) |
| G3 | 11(10.1) | 271 (10.4) |
| Follow-up(years) | | |
| Median | 5 | 3.9 |
| Maximum | 5 | 14.8 |
| Recurrence | | |
| No | 40 (36.7) | 1356 (52.2) |
| Yes | 69 (63.3) | 1240 (47.8) |
| Progression | | |
| No | 95 (87.2) | 2317 (89.3) |
| Yes | 14 (12.8) | 279 (10.7) |
| Survival | | |
| Alive | 98 (90) | 1743 (67.1) |
| Dead | 11(10) | 279 (32.9) |

**TABLE 3**
**Number of Study Group Patients According to EORTC Risk Group Scores for Recurrence and Progression and Observed Proportion of 5-Year Recurrence and Progression in the Study Population**

| Recurrence Risk Group | No. in Each Group | Patients with Recurrence | Observed 5-Year Recurrence Proportion (%) | Progression Risk Group | No. in Each Group | Patients. with Progression | Observed 5-Year Progression Proportion (%) |
|---|---|---|---|---|---|---|---|
| Ir | 2 | 1 | 50 | Ip | 27 | 0 | 0 |
| Iir | 68 | 38 | 55.9 | IIp | 53 | 3 | 5.7 |
| IIIr | 33 | 24 | 72.7 | IIIp | 26 | 10 | 38.5 |
| Ivr | 6 | 6 | 100 | IVp | 3 | 1 | 33.3 |
| Total | 109 | 69 | | | 109 | 14 | |

**TABLE 4**
**Comparing the Predicted Probabilities of Recurrence and Progression of Our Study Group with Original EORTC Algorithm**

| | No. of Patients | 1-Year Predicted Probabilities with (95% CI) (%) | | 5-Year Predicted Probabilities with (95% CI)(%) | |
|---|---|---|---|---|---|
| | | Study Group | Algorithm | Study Group | Algorithm |
| Recurrence Groups | | | | | |
| Ir | 2 | 0.0(0.0,0.0) | 15(10,19) | 100.0(100.0,100.0) | 31(24,37) |
| IIr | 68 | 40.3(28.6,52.1) | 24(21,26) | 56.0(43.9,68.0) | 46(42,49) |
| IIIr | 33 | 59.6(42.5, 76.7) | 38(35,41) | 77.7(52.5,92.9) | 62(58,65) |
| Ivr | 6 | 100.0(100.0,100.0) | 61(55,67) | 100.0(100.0,100.0) | 78(73,84) |
| Progression Groups | | | | | |
| Ip | 27 | **0.0(0.0, 0.0)** | **0.2(0,0.7)** | **0.0(0.0,0.0)** | **0.8(0.0,1.7)** |
| IIp | 53 | 1.8(0.0,5.1) | 1.0(0.4,1.6) | **5.8(0.0,12.3)** | **6.0(5.0,8.0)** |
| IIIp | 26 | **4.0(0.0,48.9)** | **5.0(4.0,7.0)** | 44.1(23.5,64.7) | 17.0(14.0,20.0) |
| IVp | 3 | 33.3(0.0,86.6) | 17.0(10.0,24.0) | 33.3(0.0,86.6) | 45.0(35.0,55.0) |

*Note*: Figures in bold print indicate that the predicted probabilities of the study group are in accordance with that of the algorithm.

## DISCUSSION

Predicting the risk of recurrence and progression in non–muscle invasive bladder cancer has been the focus of much research, with the aim of individualised management for this group of patients. Conventionally, TCC has been divided into three risk groups (low, intermediate, and high), based on prognostic factors derived from multivariate analysis[10]. More recently, (updated) nomograms have been proposed to predict the probability of progression and recurrence in patients with Ta, T1, and/or CIS bladder TCC[11], which include additional criteria such as urinary NMP22, cytology, age, and gender.

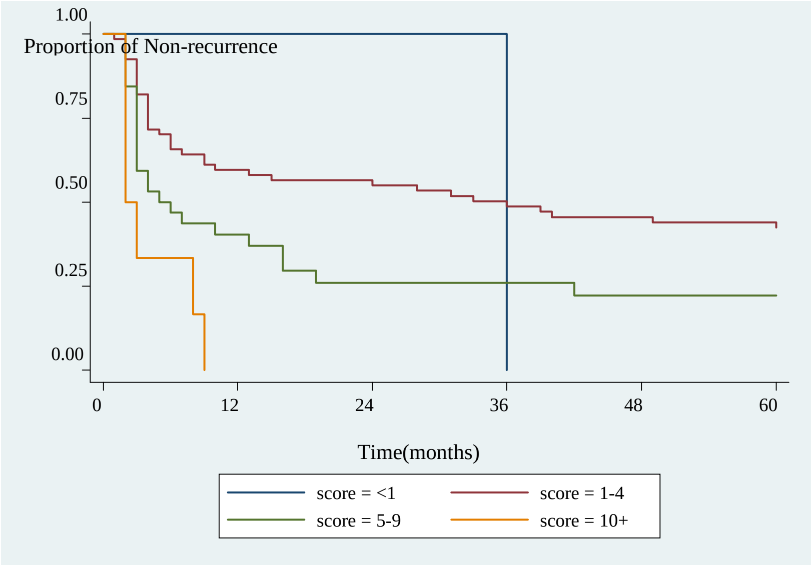

**FIGURE 1.** Time to first recurrence.

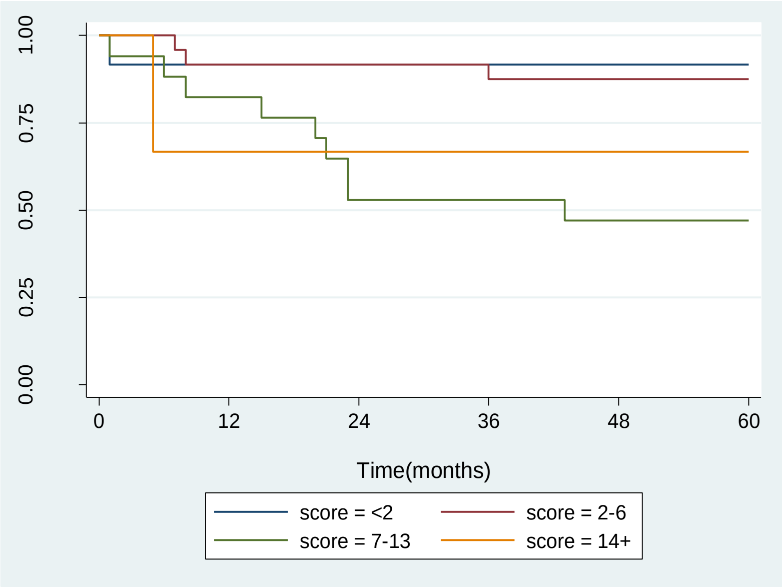

**FIGURE 2.** Time to first progression.

Sylvester's algorithm[6], derived from seven EORTC trials and incorporated into the European Association of Urology guidelines in 2006, provides simple tables to predict the risk of TCC recurrence and progression in individuals. These, however, remain to be assessed within individual institutions enabling ,for example, validity for smaller cohorts and subsets of bladder cancer populations. Our study, based on a smaller cohort of bladder cancer patients from a single institution, is the first reported study attempting to validate the algorithm.

**TABLE 5**
**Cox Model Analysis of Time to Recurrence and Progression**

| Variable | Univariate Analysis | | | | Multivariate Analysis | | | |
|---|---|---|---|---|---|---|---|---|
| | HR | 95% CI | | *p* Value | HR | 95% CI | | *p* Value |
| | | Lower | Upper | | | Lower | Upper | |
| Number of tumours (2–7 vs. single) | 1.34 | 0.45 | 3.99 | 0.6043 | 1.39 | 0.30 | 6.36 | 0.6750 |
| Number of tumours (>8 vs. single) | 5.75 | 1.92 | 17.24 | 0.0018 | 10.89 | 2.40 | 49.38 | 0.0020 |
| Tumour size (≥3 cm vs. <3 cm) | 0.40 | 0.11 | 1.45 | 0.1647 | 0.18 | 0.04 | 0.82 | 0.0262 |
| Prior recurrence (yes vs. no) | 3.41 | 0.76 | 15.28 | 0.1087 | 4.59 | 0.68 | 30.93 | 0.1176 |
| T Category (T1 vs. Ta) | 7.72 | 2.42 | 24.66 | 0.0006 | 11.60 | 2.33 | 57.72 | 0.0027 |
| CIS (yes vs. no) | 4.45 | 1.39 | 14.24 | 0.0119 | 9.13 | 1.71 | 48.77 | 0.0097 |
| Grade (3 vs. 1 and 2) | 5.70 | 1.76 | 18.40 | 0.0036 | 1.90 | 0.44 | 8.16 | 0.3868 |

HR = hazard ratio.

Some of the differences in outcome may be accounted for by differing patient characteristics (incidence of primary tumours, size, and pT category) between the two study groups (Table 2), e.g., only a small percentage of our study population received intravesical chemotherapy (19.3%) compared to Sylvester's group (78.4%). The sample size of our study population, especially the small numbers in some individual risk groups, also influenced the study results. For most of the comparisons, the confidence intervals overlap – suggesting the possibility that the nomogram may be valid even though the median numbers are widely different. In addition, the Kaplan-Meir method, while accurate for calculating 1-year predicted probabilities, might not be accurate when predicting the 5-year probabilities[12].

Our study has the advantage of examining an unselected cohort of consecutive patients with non–muscle invasive TCC as opposed to data originating from clinical trials in which patients are selected for interventions by stringent inclusion/exclusion criteria. As a single-centre study, it was possible to ensure that all bladder cancer patients within a given time period were included, reducing or avoiding selection bias that may play a role with the less exact reporting that may occur in multicentre registries. There was also a comprehensive record of tumour characteristics at initial cystoscopy in all patients, histology review by a single pathologist, and in 95% of the cases, muscle was present in the specimen, increasing accuracy of confirming pT category. All clinical parameters were collected and recorded by one person, minimising interobserver variability. Outcomes were also less likely to be influenced by intravesical chemotherapy as in the original EORTC study, a factor important when predicting prognosis, but not discussed in Sylvester's original algorithm[13,14]. Statistical methods used for predicting the probabilities were similar to those used for the original EORTC algorithm.

Although the original EORTC algorithm was intended to be universally applicable for all non–muscle invasive TCC, it cannot be applied to patients with diffuse lesions on TUR or CIS on histology, as scores could not be assigned to them for their size, numbers, or pT category and grade (with CIS). This is relevant considering that 10% of all CIS can present in a primary form without associated papillary tumour[15]. Also, the original algorithm measures two variables (tumour size and number) that are especially subjective and vary between different operators. There is also interobserver variation amongst histologists when evaluating the pT category and grade of TCC[16]. In addition, the fact that some of the progression predictions were in accordance with the original algorithm as opposed to the recurrence predictions reinforces the likelihood that recurrence and progression are influenced by additional variables other than those six selected for inclusion[17,18].

## CONCLUSIONS

The primary objective of our study was to assess the applicability of the risk calculator tool and validate it against the existing EORTC algorithm in a cohort of consecutive patients from a single centre. Although the risk calculator was user friendly, there were marked differences between the predicted probabilities of recurrences in all the risk groups and some differences in the predicted probabilities of progression when compared to actual events. The low patient numbers in individual groups limits the ability to draw firm conclusions about the validity of the nomogram, but our study does expose some of the drawbacks of standardised prognostic algorithms derived from the outcome of prospective randomised trials, when applied to local clinical practice.
